# The Efficacy of Flywheel Inertia Training to Enhance Hamstring Strength

**DOI:** 10.3390/jfmk7010014

**Published:** 2022-01-20

**Authors:** Joey O’ Brien, Declan Browne, Des Earls, Clare Lodge

**Affiliations:** 1 HealthCore, Department of Science and Health, Institute of Technology Carlow, R93 V960 Carlow, Ireland; Declanbrowne@itcarlow.ie (D.B.); desearls@itcarlow.ie (D.E.); clarelodge@itcarlow.ie (C.L.); 2 High Performance Unit, WIT Arena, X91 P20H Waterford, Ireland

**Keywords:** flywheel inertia training, hamstring strength, eccentric, inertia load

## Abstract

The purpose of this narrative review is to examine the efficacy of flywheel inertia training to increase hamstring strength. Hamstring strain injury is common in many sports, and baseline strength deficits have been associated with a higher risk of hamstring strain injury. As a result, strength and conditioning professionals actively seek additional techniques to improve hamstring strength with the aim of minimising the incidence of hamstring strain injury. One method of strength training gaining popularity in hamstring strength development is flywheel inertia training. In this review, we provide a brief overview of flywheel inertia training and its supposed adaptions. Next, we discuss important determinants of flywheel inertia training such as familiarisation, volume prescription, inertia load, technique and specific exercise used. Thereafter, we investigate its effects on hamstring strength, fascicle length and hamstring strain injury reduction. This article proposes that hamstring specific flywheel inertia training can be utilised for strength development, but due to the low number of studies and contrary evidence, more research is needed before a definite conclusion can be made. In addition, as with any training modality, careful consideration should be given to flywheel inertia training determinants. This review provides general recommendations of flywheel inertia training determinants that have value when integrating flywheel inertia training into a hamstring strengthening program.

## 1. Introduction

Hamstring strain injuries (HSI) are among the most common types of injuries seen in the area of sports medicine in elite athletes, with a prevalence of 6 to 25%, depending on the sport [1,2,3]. It is the single most frequent injury in professional football [4], rugby union [5] and track and field, especially among sprinters and jumpers [6]. Elkstrand et al., in a prospective study, [3] established that HSI accounts for 37% of muscle injuries in professional soccer players and 25% of athlete match absences. HSI has a high re-injury rate [7], with many taking place within two weeks of the original injury [8]; a rationale for this may be due to inadequate rehabilitation programs and/or a premature return to sport [9]. The function of the hamstring is complicated, as it acts as a hip extensor, knee flexor, and external rotator of the hip and knee, depending on leg positioning and connection to the ground [4]. There are two main types of acute hamstring strain mechanisms, which are best characterised by their injury settings [10]. The most prevalent type of HSI occurs during high-speed running, followed by HSI as a result of activities that cause rapid hamstring lengthening such as high kicking, sliding tackle, and sagittal split [10]. There are two views in the literature about the mechanism of hamstring injuries sustained during high-speed running. One is that the hamstring is most susceptible to damage during active lengthening, which occurs during the running gait cycle’s late swing phase [11]. However, it has also been suggested that hamstring injury occurs during the initial stance phase as the body is driven forward above the touchdown point, due to the presence of large forces acting in opposing directions [12]. Following recent reviews [13,14] it now seems to be most accepted that HSI during sprinting are most likely to occur due to excessive muscle strain caused by eccentric contraction during the late swing phase of the running gait cycle [15].

HSI’s can result in adverse effects on an individual’s performance, thereby hampering a team’s success [16]; therefore, raising the awareness of common risk factors is essential. Older age and previous injury history have been commonly associated with a higher risk of future HSI [17,18] and have been previously described as non-modifiable risk factors [19]. A topical literature review [20] highlighted numerous modifiable risk factors, including flexibility, fatigue, high speed running loads, sprint running, lumbo-pelvic hip control, insufficient/inadequate warm-up, strength and intra-limb and inter-limb asymmetry and biceps femoris fascicle length. To date, hamstring strength is the most researched, with various types of muscular strength deficits being associated with a higher risk of in HSI numerous studies [21,22,23,24,25]. As a result, strength and conditioning professionals actively seek additional techniques to improve hamstring strength to minimise the incidence of HSI with non-traditional methods of strength training such as Flywheel Inertial Training (FIT) gaining popularity. 

When FIT was first developed, it was intended to help astronauts cope with the neuromuscular dysfunctions and contemporaneous muscle atrophy that result due to the lack of gravity experienced during long-term space travel [26,27]. Many studies have since described the mechanical advantages of flywheel devices and attempted to clarify the neural and physiological mechanisms, structural adaptations and training effects induced by flywheel exercise [28]. However, few have specifically investigated it as a hamstring strength training modality. FIT is based on the flywheel principle, whereby the inertia generated by rotating flywheels provides resistance [29]. The exerted force unwinds a strap attached to the device’s shaft during the concentric phase, causing the flywheel to rotate; when the concentric phase is completed, the strap rewinds and the user must resist the device by performing an eccentric muscular action [30]. This technique can result in brief moments of eccentric overload if performed correctly [31]. This eccentric overload may induce positive gains in hamstring strength. Currently, there is a scarcity of research investigating FIT to increase hamstring strength, specifically eccentric hamstring strength. Therefore, the main aim of this review is to investigate the efficacy of FIT to increase hamstring strength while also investigating determinants of hamstring specific FIT to provide practical guidelines to be used in hamstring specific FIT. 

## 2. Materials and Methods

The following search strategies were used to locate relevant articles online. Databases searched included EBSCO host, Web for Science, PubMed, Pub Med, SPORTDiscus and Google Scholar. Relevant key terms in the search included training flywheel inertia training OR flywheel device OR rotational inertia device OR inertia load AND hamstring strength AND eccentric strength. Additionally, articles cited in the reference lists of acknowledged journals were manually searched and examined.

### 2.1. Familiarisation

To fully benefit from FIT, a certain amount of coordination is required [32], thus experience with these devices will have an impact on the results gained, especially if the exercises entail movements in a closed kinetic chain where the displacement is done against gravity [33]. Familiarisation with FIT could alter how the concentric and eccentric phases of an exercise are performed, which may, in turn, alter the eccentric overload stimulus experienced [34]. For example, Tous-Fajardo et al. [32] reported that participants with experience in flywheel training achieved a higher eccentric overload when compared to participants with no previous experience. Twenty male field sport athletes took part in the study. Ten participants had previous experience (>5 sessions) in the flywheel leg curl exercise, while ten with similar physical characteristics and training history had only experienced <2 familiarisation sessions. Athletes with more FIT experience had considerably higher eccentric peak forces than concentric peak forces (<0.05); additionally, athletes of the same ability who were familiar with FIT generated greater eccentric and concentric peak force. It appears that a certain amount of coordination is required to apply braking forces at the end of the eccentric action, which is warranted to elicit an eccentric overload; fine-tuning this strategy appears to require adequate familiarisation [32]. 

Piqueras-Sanchiz et al. [35] reported high (0.7−0.89) to very high (>0.9) reliability during both concentric and eccentric power production over varying inertial loads. The authors tested both males and females across four testing sessions in the flywheel leg curl exercise using varying inertial loads (0.083, 0.132, 0.182, 0.266 and 0.350 kg·m^2^). An increase in power reliability was established across all testing sessions with very high reliability (>0.9) scores for all inertial loads in session four. Both males and females presented the highest reliability scores in the fourth session, prompting authors to conclude that to achieve reliable and stable outputs, a familiarisation process with the flywheel leg curl exercise is required with inexperienced subjects, recommending two to four sessions depending on the training experience. Less experienced subjects may need more extended familiarisation periods. 

Similar research [36] conducted during the flywheel squat exercise observed comparable reliability scores (0.79−0.93). The authors in that study recommended performing at least three sessions to obtain a stable measure during the flywheel squat exercise. A more recent [33] study examined the differences in kinetic and kinematic profiles between two different FIT devices. A half-squat incremental test was performed by 39 healthy males on two different FIT devices: one a horizontal cylinder and the other a vertical cone-shaped axis. The study reported differences in biomechanical output between athletes with different levels of experience in the use of FIT; peak force during the ECC phase was delayed with respect to the start of the next CON phase in the less experienced group. This delay may affect the amount of force that can be produced in the ECC phase. This agrees with previous research [32], which established that to apply maximal force towards the end of the ECC stages of movement, a certain amount of co-ordination is required. Research suggests that this co-ordination is best achieved by FIT experience. The differences described in this study [33] were consistent in both FIT devices analysed. 

The research definitively shows that a familiarisation period is warranted to obtain reliable, stable measures in flywheel training (two to four sessions). It may be conceivable that less complex exercises such as a flywheel leg curl may require shorter familiarisation periods when compared to more complex exercises such as flywheel Romanian deadlifts, but more research is required to make a final determination. It is crucial to understand how FIT variables such as power output production and eccentric overload are affected by participant familiarisation.

### 2.2. Inertial Load

An important determinant that can affect FIT performance is inertial loading. Depending on the inertial load used, different mechanical responses have been observed, which appears to be an essential factor in optimising training results [36]. Tous-Fajardo et al. [32] was the first study to investigate the effects of inertial load in a hamstring specific leg curl exercise. Twenty male amateur soccer and rugby players performed one testing session, which consisted of two sets of six-coupled concentric–eccentric actions. Each set used two different inertial loads (0.11 and 0.22 kg·m^2^). The larger inertia showed greater peak force, although it should be noted that the inertial loads used would be classed as low inertial loads in comparison to more recent similar research. 

Piqueras-Sanchiz et al. [35] also investigated the effect of inertial load on power output in a flywheel leg curl. Sixteen amateur university sports athletes participated in the study. The study evaluated the influence of different inertial loads (0.083, 0.132, 0.182, 0.266, and 0.350 kg·m^2^) on concentric power, eccentric power, and eccentric overload ratio (eccentric peak power/concentric peak power ∗ 100) using a parallel-group design. Both males and females (*n* = 16) took part in the intervention. The study discussed that manipulating the inertial load can modify the power generated, with lower inertial loads (0.083 and 0.132 kg·m^2^) leading to higher concentric and eccentric power output; however, there was no significant change (<0.05) in eccentric overload between inertial loads. Males did, however, show a more significant difference from the lowest to the higher inertia (ES = 1.37 to 1.51) than females (ES = 1.41 to 1.47), with the authors attributing this to lower strength levels of the female participants, meaning they used a higher relative load and may have a greater resistance to fatigue at higher relative intensities [35]. These findings are not in line with previous research [36,37] which showed that larger inertial loads achieved higher eccentric overloads; however, the studies mentioned both investigated the flywheel squat exercise, so the research may not be comparable. 

A more recent hamstring specific intervention [38] investigated the effects of inertial load in a flywheel Romanian deadlift. Fourteen recreationally trained males partook in the study, and they all had a minimum of two years of resistance training experience. No participant had FIT experience; however adequate familiarisation was provided. The inertial loads ranged from low to high (0.025, 0.050, 0.075, and 0.100 kg·m^2^). Results again highlighted that a lower inertial load led to higher peak concentric and eccentric peak power outputs, with the lowest load achieving the highest value (0.025 kg·m^2^). Contrary to Piqueras-Sanchiz et al. [35], medium to higher loads (0.050, 0.075, and 0.100 kg·m^2^) led to higher eccentric overload output, with 0.100 kg·m^2^ displaying the largest value. The authors proposed that single-joint exercises such as a flywheel leg curl might recruit fewer muscles than multiple joint exercises such as a flywheel RDL; participants may not have been able to fully resist and break inertial forces, which could lead to a notable decline in eccentric overload. The most effective technique to maximise eccentric overload is to gently resist the force during the first third of the eccentric phase, then maximally decelerate the rotating flywheel and stop at the end range of motion [39]. Research may differ on the effects of higher inertial loads; but it is irrefutable that lower loads result in higher peak power outputs. This has significant practical implications because it may lead to greater performance adaptations. As previously discussed, the load that maximised power output may be the most efficient to develop athletic performance [40,41,42].

### 2.3. Volume Prescription

Intensity (inertial load) plays a vital role in training prescription during FIT, but it is matched in importance by volume (number of repetitions used), as it is vital to any training intervention aiming to achieve a specific adaption. To the author’s knowledge, no study to date has analysed volume prescription for any hamstring specific FIT exercise. One study [36], however, using a flywheel bilateral squat, investigated the number of repetitions using different inertial loads at which concentric and eccentric peak power maintenance is observed. Participants performed one set of 15 repetitions at varying inertial loads (0.025, 0.050, 0.075 and 0.100 kg·m^2^), with each load being tested on a different day over four testing days. The highest value was not found in the first two repetitions for both concentric and eccentric peak power; as in FIT, the first two repetitions are generally used to build momentum of the flywheel. Therefore, the authors recommended that three to four repetitions are warranted to accelerate the flywheel at the start of the set, allowing a build-up to maximal effort. Depending on the inertial load, a range of five to 12 repetitions was advised to maintain power output, but the authors concluded that it is essential to individualise the training volume prescription due to inter-subject variability in power decrements. Previous hamstring specific studies have used a fixed number of repetitions [43,44,45], or have increased the number of repetitions throughout the intervention [46,47], but no clear criteria for volume prescription has been advised. This highlights that more research into volume prescription is warranted, specifically with regard to the number of repetitions used in hamstring specific FIT, as it is commonly regarded that different training volumes lead to specific neuromuscular adaptations [36] and need to be targeted primarily. 

### 2.4. FIT Technique and Exercise Used

According to previous research [39], the most effective method for providing an eccentric stimulus includes gently resisting the inertial force during the first third of the eccentric action and then exerting total effort to decelerate the revolving flywheel and bring it to a halt after the eccentric phase of the exercise. The subsequent concentric phase is then completed with maximal effort, and the discussed eccentric phase-specific technique is repeated. It should be highlighted that participants may not always adopt this technique [34]. Individuals may attempt to control the velocity of the eccentric phase in anticipation of the more challenging braking stimulus at the end of the movement, which would lead to a decreased eccentric overload [39], perhaps due to an involuntary self-protection mechanism in an effort to avoid peak forces [32]. Avoiding peak forces at the end range may be counterproductive, especially in hamstring training, as this end range is where the hamstrings are most susceptible to injury. In the flywheel leg curl, Tous-Fajardo et al. [32] discovered the opposite, that the window where the more substantial eccentric force was generated occurred in the later stages of a range of motion. Given the complexity of FIT, auto-regulated feedback could aid in readapting performance and getting the athlete comfortable with the correct technique. 

Exercise selection may alter the stimulus provided during FIT; it has been previously hypothesised [36] that the number of muscles recruited during FIT may affect performance. Large multi-joint movements such as an RDL may allow participants to halt higher inertial loads more effectively and therefore achieve more significant eccentric overload at these high loads compared to single multi-joint movements such as a leg curl which recruits less musculature. Exercise selection may also affect the musculature that is predominantly used. For example, the flywheel leg curl has been previously shown to recruit the medial hamstring muscles [48] preferentially; hip-dominant exercises (such as an RDL) preferentially recruit the BFlh [49], which is the most frequently injured muscle of the hamstring complex [50]. It may be beneficial to target the most frequently injured muscle, but to the authors’ knowledge, no study has directly compared a flywheel hip dominant exercise to a knee dominant exercise regarding hamstring adaptations. Further research is needed before any conclusions can be made. 

### 2.5. Efficacy of FIT on Hamstring Strength Development

In sports with high intensity running demands, such as soccer [50] and Australian rules football [24], eccentric hamstring weakness has been identified as a risk factor for future HSI. Timmins et al. [50] assessed the eccentric hamstring strength of 152 professional soccer players in a sizeable prospective study. Players with eccentric hamstring strength below 4.35 N·kg^−1^ were 4.4 times more likely (RR; 95% CI 1.1 to 17.5) to sustain an HSI in the following season than stronger players. In agreement with this, Opar et al. [24] found that eccentric strength below 256 Newtons (N) at the start of pre-season and 279 (N) at the end of pre-season were said to associate with an increased risk of HSI (2.7- and 4.3-fold, respectively) in a population of 210 elite Australian footballers. Such research highlights the importance of eccentric hamstring strength in HSI and may explain why it is so prevalent in injury prevention programs and in the research. Flywheel inertia training has grown in popularity in recent years, and if performed appropriately, can result in moments of eccentric overload. This eccentric overload may have the potential to improve hamstring strength and warrants investigation.

Askling and colleagues [43] assessed isokinetic concentric and eccentric strength in elite-level male soccer players (*n* = 30). Players were randomly assigned to an intervention (*n* = 15) and to a control group (*n* = 15). Both groups followed the same training regime, with the intervention group performing extra-specific hamstring-specific FIT. The intervention group performed 16 sessions which included four sets of eight repetitions of a flywheel leg curl; the first set was submaximal and used as a warm-up. The training session fell on every fifth day for the first four weeks, then every fourth day for the last six weeks, lasting ten weeks in total. The intervention group showed a significant (<0.05) increase in both concentric and eccentric knee flexor peak torque, with the control group exhibiting no meaningful change. The increase in both concentric and eccentric strength was similar, 15 and 19%, respectively. The study suggested that eccentric overload training may cause greater hamstring strength adaptions than concentric training on its own. It should be noted that there were no familiarisation sessions held during the training period. This is significant because, as previously stated, 2–4 familiarisation sessions are recommended before adequate technique is achieved. The first sessions of the study may have only gained participant experience with the device and not actually achieved the desired strength training stimulus. 

Timmins et al. [47] compared the effects of a flywheel RDL to an NHE on eccentric hamstring strength in elite-level Australian footballers. Twenty-seven male athletes were randomised into two groups, and the intervention took place over a 39-week period which included both pre-season and in-season. Eccentric hamstring strength was assessed at three-time points: baseline, end of pre-season and the end of the intervention. To create an appropriate stimulus, both training programs were periodised by gradually increasing the volume and additional weight employed throughout training, with both groups being matched for volume and intensity. The NHE group were required to use a weight (5–10 kg) to achieve desired intensity, whereas the RDL group used 0.05 or 0.075 kg·m^2^. The intervention showed favorable adaptions for both groups, with both groups increasing their eccentric strength (RDL: mean change 82N, 95%CI 12 to 152N, d = 1.34, *p* = 0.026; NHE: mean change 97N, 95%CI 47 to 146N, d = 1.77, *p* = 0.001). As previously discussed, having eccentric strength values of 279N at the close of pre-season elevated the chance of a future HSI four times in professional Australian Football [24]. Timmins et al. [47] concluded that the eccentric hamstring strength adaptions gained in both groups from the intervention might positively impact HSI risk reduction. This was the first study to investigate a flywheel hip dominant exercise on modifiable risk factors of HSI, and although the results were favorable, more research is necessary. 

Contrary to the previously mentioned studies [43,47], Presland et al. [51] found no significant increase in eccentric hamstring strength following a six-week flywheel leg curl protocol. Participants were randomised into two groups, a control (*n* = 10) and an eccentric biased group (*n* = 10). The control group engaged in unilateral training on the flywheel device with their opposite leg acting as a non-exercising control leg. The eccentric biased group also engaged in flywheel training. They performed the concentric phase with both legs but only one leg when undertaking the eccentric phase. The same leg was used throughout the training intervention. Eccentric strength was assessed pre and post-intervention using a previously validated NHE field testing device (NordBord, Vald Performance, Queensland, Australia) [52]. Although no statistical significance increases were reported, some increases were found among both groups, ranging from 33N to 46N. The study discussed possible neural adaptations in the control limbs contributing to these changes in eccentric strength, but this is unclear and needs further investigation. It should be noted that different flywheel exercises and protocols were used compared to previous research [47], so the direct comparison may be unfair. Research indicates that hamstring specific FIT can be utilised for strength development [43,47], but due to the low number of studies and contrary evidence [51], more research is needed before a definite conclusion to be made. 

### 2.6. Fascicle Length

Hamstring strains comprise 37% of all muscle strain injuries, and of those, the majority occur in the biceps femoris long head (BFlh) [50]. Despite a paucity of evidence, it has been suggested that the length of the hamstring muscle fascicle may influence the likelihood of a future HSI [53,54], specifically the BFlh. One previous study [55] found BFlh fascicles to be shorter in previously injured muscles when compared to contralateral uninjured muscles. Adding weight to this concept, Timmins and colleagues [50] revealed that athletes who suffered a previous HSI possessed shorter BFlh fascicles than their uninjured counterparts while adding that non-modifiable risk factors such as age and previous hamstring injury were negatively influenced by shorter BFlh fascicles concerning HSI risk. They concluded that short BFlh fascicles were associated with an increased risk of future HSI in elite soccer players. Shorter fascicles, with fewer in-series sarcomeres, were previously thought to be more prone to being overstretched and damaged by severe eccentric contractions, such as those experienced during the terminal swing phase of high-speed running [53] but this is still debated in the research. Even if the exact mechanism of why shorter fascicle length increases the risk of HSI is unclear, it is commonly regarded that increasing fascicle length, specifically in the BFlh, is a worthwhile venture. Previously, the Nordic hamstring exercise was the most used tool for this [56], but recently FIT has gained interest as a modality to enhance BFlh fascicle length. 

Two previously discussed studies [47,51] were in disagreement on FIT for enhancing eccentric hamstring strength, but agreed on its ability to increase BFlh fascicle length. Timmins et al. [47] found increases in both a flywheel RDL group and an NHE group (RDL: d = 1.99, *p* < 0.001; NHE: d = 1.73, *p* < 0.001) when compared to pre-intervention assessments, with the RDL grouping showing a slightly larger effect. This may be partly because hip dominant movements, such as an RDL, preferentially recruit the BFlh compared to knee dominant ones [57]. Presland et al. noted that after six-weeks of training, subjects who did the eccentrically biased flywheel intervention had a significant 14 ± 5% (*p* < 0.001, d = 1.98) increase BFlh fascicle length. It should be noted that these adaptions declined after a four-week detraining period, which highlighted that continuing eccentric loading is essential for the maintenance of architectural adaptations following flywheel leg-curl training. The lengthening of BFlh fascicles has been hypothesised [58] as playing a role in the effectiveness of eccentric training programs in reducing the risk of future HSI. If correct, then the discussed studies reflect positively on specific hamstring FIT as a modality to decrease HSI risk by positively affecting fascicle length, specifically BFlh. 

### 2.7. HSI Injury Risk Reduction Using FIT

Strength training has been advocated for preventing HSI for many years [59] and forms the basis of most injury prevention programs used today. Askling and colleagues [43] were the first to investigate whether a flywheel strength training program, which emphasised eccentric loading using the leg curl exercise, might affect the occurrence and severity of HSI. Over the ten-week intervention, players from two elite level Swedish soccer teams were randomised into two groups. One group performed specific hamstring FIT (flywheel leg curl), while the control group did not. Six in the intervention group and four in the control group reported an HSI in the previous season. All injuries were recorded during the intervention period, and an injury was included if it occurred during a match or training session and if a player missed a minimum of one match or training session. A significantly (<0.05) lower number of injuries was reported in the intervention group. The authors noted that the positive effects shown in the study advocated the use of hamstring specific FIT in elite soccer but did not fully attribute the preventive effect on HSI to the eccentric overload derived from FIT and suggested that further longitudinal studies are needed for more definite recommendations. 

De Hoyo et al. [44] again investigated the effects of FIT eccentric overload training in elite soccer players, but this time in junior players. Thirty-six elite-level junior (U17-19) players were divided into two groups (intervention v control). The intervention group performed 1-2 FIT sessions weekly, including a flywheel half squat and leg curl exercise. Both groups performed the same volume of match play and training sessions over the ten-week intervention. The control group performed no strength training during the entire season. The number of training sessions and matches and the number of muscle injuries per 1000 h of exposure (match and training) were recorded. The study reported an overall reduction of injury following training. The study monitored all injuries, not just hamstring ones. This fact should be considered when disseminating the findings. Considering that the protocol included a flywheel squat exercise, it cannot be determined how much of a role the hamstring specific exercise (leg curl) played in the positive findings. Nevertheless, it can be agreed that FIT in general, regardless of exercise, may be incorporated into injury reduction strategies.

## 3. Practical Guidelines and Discussion

- To maximise the benefits of FIT, it is advised to first perform several familiarisation sessions. Two to four familiarisation sessions are recommended to obtain a stable measure. Less complex exercises such as a flywheel leg curl may require shorter familiarisation periods when compared to more complex exercises, and more experienced athletes may require shorter familiarisation periods than novice athletes.
- Lower inertial loads (0.025 kg·m^2^) lead to higher peak concentric and eccentric peak power outputs, while medium to higher loads (0.050, 0.075, and 0.100 kg·m^2^) lead to higher eccentric overload output.
- Although there is no hamstring-specific research available, flywheel squat research suggests that a range of five to 12 repetitions was advised to maintain power output depending on the inertial load. Three to four repetitions are warranted to accelerate the flywheel at the start of the set, to build to maximal effort, and should be viewed as waste repetitions and not counted as working repetitions. Due to large subject variability in FIT, it is essential to individualise the training volume prescription.
- Both the flywheel leg curl and RDL have been shown to increase eccentric hamstring strength in elite level athletes, but more research is needed before a definite conclusion can be made.
- Hamstring specific FIT has been shown to increase fascicle length, specifically in the BFlh. Thus, this architectural adaption could help with a risk reduction of future HSI.
- The most effective technique to achieve an eccentric overload comprises gently resisting the inertial force during the first third of the eccentric action and then exerting a full effort braking action to decelerate the revolving flywheel and bring it to a halt at the end range of motion.
- The flywheel leg curl has been shown to affect the reduction of injuries in elite soccer players positively, but more longitudinal studies are warranted. It may also be interesting to investigate other FIT hamstring specific exercises such as the RDL as injury prevention techniques.

Overall, hamstring specific FIT has positive adaptions on hamstring strength and other modifiable HSI risk factors, such as fascicle length. It should be highlighted that there was a dearth of research on these themes accessible for review, and this fact should be considered when processing these conclusions. To gain the desired stimulus from FIT, its determinants should also be considered. This review highlights that athlete familiarisation and inertia load may both influence the adaptations of FIT and should be monitored carefully. Although there is positive research available regarding volume prescription and varying exercises, more is warranted to investigate different hamstring specific exercises over extended intervention periods. This narrative review is not intended to be a definitive guide for FIT, but it may help practitioners implement best practices while using this method to enhance hamstring strength.

## Data Availability

Data will be made available upon reasonable request to the corresponding author.
